# Determinants of undisclosed HIV status to a community-based HIV program: findings from caregivers of orphans and vulnerable children in Tanzania

**DOI:** 10.1186/s12981-020-00299-8

**Published:** 2020-07-16

**Authors:** John Charles, Amon Exavery, Asheri Barankena, Erica Kuhlik, Godfrey M. Mubyazi, Ramadhani Abdul, Alison Koler, Levina Kikoyo, Elizabeth Jere

**Affiliations:** 1 Pact, P.O. Box 6348, Dar es Salaam, Tanzania; 2 Ifakara Health Institute, P.O. Box 78373, Dar es Salaam, Tanzania; 3 Pact, Inc., 1828 L St NW Suite 300, Washington, DC 20036 USA; 4 National Institute for Medical Research (NIMR), P.O. Box 9653, Dar es Salaam, Tanzania

**Keywords:** HIV, Disclosure, Child, Orphan, Caregivers, Kizazi Kipya, Tanzania

## Abstract

**Background:**

HIV status disclosure facilitates receipt of HIV prevention and treatment services. Although disclosure to sexual partners, family members or friends has been extensively studied, disclosure to community-based HIV programs is missing. This study assesses the magnitude of, and factors associated with undisclosed HIV status to a community-based HIV prevention program among caregivers of orphans and vulnerable children (OVC) in Tanzania.

**Methods:**

Data are from the USAID-funded Kizazi Kipya project that seeks to increase uptake of HIV, health, and social services by OVC and their caregivers in Tanzania. Data on OVC caregivers who were enrolled in the project during January–March 2017 in 18 regions of Tanzania were analyzed. Caregivers included were those who had complete information on their HIV status disclosure, household socioeconomic status, and sociodemographic characteristics. HIV status was self-reported, with undisclosed status representing all those who knew their HIV status but did not disclose it. Multilevel mixed-effects logistic regression, with caregivers’ HIV status disclosure being the outcome variable was conducted.

**Results:**

The analysis was based on 59,683 OVC caregivers (mean age = 50.4 years), 71.2% of whom were female. Of these, 37.2% did not disclose their HIV status to the USAID Kizazi Kipya program at the time of enrollment. Multivariate analysis showed that the likelihood of HIV status non-disclosure was significantly higher among: male caregivers (odds ratio (OR) = 1.22, 95% confidence interval (CI) 1.16–1.28); unmarried (OR = 1.12, 95% CI 1.03–1.23); widowed (OR = 1.12, 95% CI 1.07–1.18); those without health insurance (OR = 1.36, 95% CI 1.28–1.45); age 61 + years (OR = 1.72, 95% CI 1.59–1.88); those with physical or mental disability (OR = 1.14, 95% CI 1.04–1.25); and rural residents (OR = 1.58, 95% CI 1.34–1.86). HIV status non-disclosure was less likely with higher education (*p *< 0.001); and with better economic status (*p *< 0.001).

**Conclusion:**

While improved education, economic strengthening support and expanding health insurance coverage appear to improve HIV status disclosure, greater attention may be required for men, unmarried, widowed, rural residents, and the elderly populations for their higher likelihood to conceal HIV status. This is a clear missed opportunity for timely care and treatment services for those that may be HIV positive. Further support is needed to support disclosure in this population.

## Background

HIV status disclosure is vital in HIV prevention and treatment programs as a tool for prevention and care strategies [1–6]. In 2014 at the 20th International AIDS Conference in Melbourne, Australia, the United Nations Programme on HIV and AIDS (UNAIDS) launched the 90–90–90 targets for HIV and AIDS programming. These targets state that by 2020, 90% of all people living with HIV will know their status; 90% of people diagnosed with the HIV infection will be on antiretroviral therapy (ART); and 90% of people on treatment will be virally suppressed [7]. The 90–90–90 goals intended to stimulate national and global action to control HIV and end the AIDS epidemic by 2030 [7, 8]. Massive gains in the realization of these targets largely depends on the success of the first 90 in the cascade. However, recent estimates show that in 2018, 79% of all people living with HIV globally knew their status [9], while in Tanzania this was estimated at 60.6% in 2017 [10].

It has been reported that the process of disclosure of one’s HIV status to sexual partners, family, and friends is complex, with potential for both positive and negative consequences [11, 12]. For instance, disclosure may result in increased stigma, discrimination, partnership dissolution [13, 14], blame, and domestic violence [13]. In fact, stigma has been cited as the largest stumbling block for HIV status disclosure [15–17]. However, disclosure has also been shown to lead to increased social support and intimacy with partners, reaffirmation of one’s sense of self, and the opportunity to share personal experiences and feelings with sexual partners [14]. Furthermore, HIV status disclosure has been associated with higher rates of adherence to antiretroviral therapy (ART) among people living with HIV (PLHIV) [18]. HIV status disclosure may lead to improved access to HIV prevention and treatment services as well as increased opportunity for risk reduction and increased opportunities to plan for the future [19]. Mathematical modelers have shown that there is up to 41% reduction in HIV transmission attributable to serostatus disclosure [20]. Given the value, yet difficulty, of HIV status disclosure, appropriate counseling and support interventions provide significant assistance to PLHIV to disclose their status.

Global rates of HIV status disclosure among adult sexual partners vary greatly [21–23], and tend to be consistently high in high-income countries, while showing greater variation in developing countries [24]. One study reported a disclosure rate of only 17% in developing countries, compared to a rate of 86% in developed countries [6]. Another study reported that rates of disclosure among partners in sub-Saharan Africa also vary greatly [25]. For example, one study in Ethiopia found that HIV status disclosure to sexual partners was 57.4% [26] and another estimated it at 82.5% [27]. This was 84% in Kenya [28], 85.4% in Uganda [29], 62.0% in Nigeria [30, 31], and 72.4% in Mozambique [32]. Similarly, disclosure estimates among partners in the general population in Tanzania are also variable, e.g. 93.3% in Mwanza [33], 66% in Kilimanjaro [5], 56.3% in Kisarawe [34], and 28% in Morogoro [25]. These variations in disclosure rates suggest variations in contexts and a need for further research and context-specific interventions.

Many factors positively and negatively associated with HIV status disclosure have been identified including stigma and discrimination [15–17], knowledge of partner’s HIV status and membership in HIV and AIDS control associations [6], economic status [35, 36], literacy [37], gender [24], age [38–40], marital status [5, 38–40], being on ART, contraceptive use [5], and many others [25, 26, 33, 41–43].

However, most of these studies have analyzed disclosure mainly focusing on the reveal of HIV test results to one’s sexual partner, family or friends [5, 12, 38, 44–49]. Parents, guardians or caregivers’ disclosure of HIV status to infected children has been studied as well [50–53]. These studies have relied on HIV positive individuals. Only one study of the HIV status disclosure among both HIV positive and HIV negative respondents was found [48]. However, the disclosure of individuals’ positive or negative HIV status to community-based HIV prevention and treatment programs is missing in the literature. For community-based programs that support HIV prevention and treatment, disclosure of one’s HIV status is a prerequisite for provision of status-appropriate HIV services. Because HIV status disclosure is voluntary in community-based programs, non-disclosure of one’s HIV status limits the program’s ability to identify individual HIV needs and provide targeted HIV services, which maximize HIV outcomes for both negative and positive people. Therefore, disclosure support is critical to ensure linkage to appropriate HIV related services and remains at the core of the HIV epidemic control strategies.

This study assessed the magnitude of, and factors associated with undisclosed HIV status to the community-based HIV prevention and treatment program, USAID Kizazi Kipya, among caregivers of orphans and vulnerable children (OVC) in Tanzania. Since the USAID Kizazi Kipya project targets households affected or infected by HIV, the target population already has both a high burden of HIV as well as risks for HIV infection. So, it is important for the program to know their status, and this is an ideal population for conducting this kind of study. Pact is working in partnership with Elizabeth Glaser Pediatric AIDS Foundation (EGPAF), the Aga Khan Foundation (AKF), Railway Children Africa (RCA), the Ifakara Health Institute (IHI), and high-performing local civil society organizations (CSOs) to implement the USAID Kizazi Kipya project.

## Methods

### Data source

Data are from the community-based, USAID-funded Kizazi Kipya project in Tanzania. The project (2016–2021) seeks to increase the uptake of HIV, health, and social services by OVC and their caregivers. Based on self-reports of OVC caregivers, the data were collected by Lead Case Workers (LCWs) and Community Case Workers (CCWs) during beneficiary screening and enrollment using the project’s screening and enrollment, and Family and Child Asset Assessment (FCAA) tools under the direct supervision of the respective Civil Society Organizations (CSOs). Before screening and enrollment, the CSOs and the case workers were trained on the data collection tools and field logistics by the USAID Kizazi Kipya project monitoring and evaluation (M&E) staff. At the field level, all households which were screened and identified as eligible for the USAID Kizazi Kipya project were enrolled after consenting. Screening and enrollment data were captured on hardcopy questionnaires by the LCWs and CCWs after which the completed questionnaires went to the CSO office for review to ensure accuracy and completeness. The CSOs’ M&E staff were responsible for ensuring that the data met the required quality standards. The data were entered into a centralized database on tablets using a CommCare application by CSO-based data clerks.

**Fig. 1:**
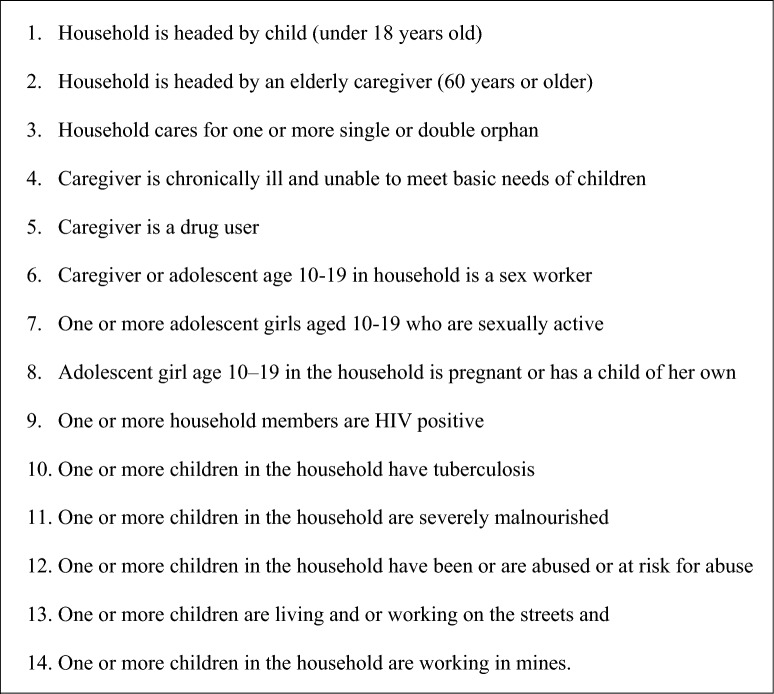
Household enrollment criteria for the USAID Kizazi Kipya project

LCWs and CCWs are lay volunteers recruited by a government standard and trained in basic social welfare case management skills. Known status or HIV positive status is not a requirement for program eligibility. Beneficiaries are enrolled into the USAID Kizazi Kipya project if their household meets one or more of the 14 household vulnerabilities related to HIV which are published [54] and also summarized in Fig. 1.

From these criteria, the OVC under the caregiver’s care in the OVC project do not necessarily have to be HIV positive. These criteria are equally applied for all councils and age groups.

After enrollment, the USAID Kizazi Kipya project provides or links caregivers as well as children and adolescents to services in the areas of health, HIV, nutrition, education, child protection, social protection, and economic strengthening. The project provides psychosocial support, nutrition assessments, counseling and support, referrals and linkages, and care plan monitoring. Through these services, HIV status disclosure support is also provided to all enrollees of unknown status at enrollment. Ultimately, the project expects to know HIV status of all its beneficiaries to enhance services coverage.

### Study area and population

Data for this study originated from 18 regions of Tanzania where the USAID Kizazi Kipya project had implemented enrollment activities during January–March 2017. These regions and their corresponding HIV prevalence in parenthesis were: Dar es Salaam (4.7%), Dodoma (5.0%), Geita (5.0%), Iringa (11.3%), Kagera (6.5%), Katavi (5.9%), Mbeya (9.3%), Mjini Magharibi (0.6%), Morogoro (4.2%), Mtwara (2.0%), Mwanza (7.2%), Njombe (11.4%), Pwani (5.5%), Rukwa (4.4%), Ruvuma (5.6%), Singida (3.6%), Tabora (5.1%), and Tanga (5.0%) [8]. From these regions, a total of 59,683 OVC caregivers who were enrolled in the USAID Kizazi Kipya project during January–March 2017 with information on their HIV status disclosure, household socioeconomic status, and sociodemographic characteristics were included. A caregiver is defined by the USAID Kizazi Kipya project as a guardian who has the greatest responsibility for the daily care and rearing of one or more OVC in a household [55]. A caregiver is not necessarily a biological parent of the OVC.

### Study design

The study design constituted a cross-sectional secondary analysis of existing monitoring data as described above. FCAA data were collected by CCW volunteers during screening of potential beneficiaries. After this, beneficiary households meeting enrollment criteria and consented, were enrolled in the program. The FCAA tool was administered in *Kiswahili*, the national language of Tanzania. The tool captured information for both the caregivers and the OVC. Under the caregivers’ section, the tool captured their demographic information, household assets, sources of income, HIV status, food security, and use of and adherence to antiretroviral therapy (ART) for those who reported their HIV status as positive. After enrollment, beneficiaries were followed up by the project over time with a variety of health and social services.

### Data analyses

Data analysis was conducted using version 14.0 of Stata statistical software. Exploratory analysis was conducted through one-way tabulations to obtain distributional features of the caregivers in each variable. Cross-tabulation of undisclosed HIV status by each of the independent variables was conducted to assess how it varied by levels of each independent variable. The Chi-Square (χ^2^) test was used to assess the degree of association between undisclosed HIV status and each of the independent variables with *p*-values reported.

Multivariate analysis was conducted using random-effects logistic regression model due to hierarchical or clustered structure of the data [56]. The usual assumption of independence of the observations did not hold since characteristics of caregivers who reside in the same community may be correlated in relation to HIV status disclosure. Thus, a multilevel model, which recognizes these data hierarchies and allows for residual components at each level in the hierarchy, was used [57]. This choice was based on the assumptions that caregivers who reside in the same community are dependent in their behavioral, physical or mental characteristics because of closer social interactions. All statistical inferences were made at a significance level of 5% (α = 0.05), whereby all estimates corresponding with a *p*-value less than 0.05 were considered statistically significant.

### Variables

HIV status disclosure at enrollment was the outcome or the dependent variable, which was derived from the caregiver’s self-reported HIV status to the program. All those who reported their HIV status as negative or positive were grouped together as having disclosed their status. Those who knew their status but refused to disclose it were referred to as not having disclosed their status. Caregivers who reported that they have never tested for HIV were excluded from the analysis. For computational purposes, the final dependent variable was referred to as undisclosed HIV status which was binary as follows;

$${\text{Undisclosed }}\;{\text{HIV }}\;{\text{status}} = \left\{ {\begin{array}{*{20}c} { 0\; if\; the\; caregiver\; DISCLOSED \;their \;HIV\; status\; to\; the \;program \;at\; enrollment } \\ {1 \;if \;the\; caregiver\; DID\; NOT\;DISCLOSE\; their\; HIV \;status \;to\; the\; program \;at \;enrollment} \\ \end{array} } \right.$$

Several independent variables were included. These were sex, age, education, marital status, mental or physical disability status, whether some or all the family members are covered by a health insurance, household wealth quintile, food security, and type of residence (rural or urban). Rural residence included all those living in district councils, whereas those living in township, municipal or city councils were considered as urban residents.

Disabilities were assessed through observations. Visually, the LCW or CCW assessed the caregiver and noted any observable disabilities and limitations (e.g. blind, mentally challenged, etc.). Health insurance in this study referred to Community Health Fund (CHF) and ‘Tiba kwa Kadi’ (TIKA). Introduced in Tanzania in 2001 to improve access to health services while protecting individuals from catastrophic health expenditures, CHF is a form of voluntary community-based health insurance for the rural informal sector [58]. TIKA was introduced in 2009 and operates in the same way as CHF, except that it focuses on urban settings [59]. CHF/TIKA membership is based on a household as an enrollment unit [59, 60], with annual premium rates ranging from US$ 4.2 to US$ 12.7 depending on the respective Local Government Authority (LGA) [61].

Wealth quintile was constructed using principal component analysis (PCA) of household assets to determine household socio-economic status [62]. Five wealth quintiles were formed, ranging from the lowest quintile (Q1) for the poorest households, to the highest quintile (Q5) for the most well-off households. Family-owned assets included in the PCA were, dwelling materials (brick, concrete, cement, aluminium and/or other material), livestock (chicken, goats, cows, and others), transportation assets (bicycle, motorcycle/moped, tractor, motor vehicle, and others), and productive assets (sewing machine, television, couch or sofa, cooking gas, hair dryer, radio, refrigerator, blender, oven, and others).

### Ethical considerations

This study received ethics approval from the Medical Research Coordinating Committee (MRCC) of the National Institute for Medical Research (NIMR) in Tanzania with certificate number NIMR/HQ/R.8a/Vol.IX/3024 issued on 27th February 2019. Since validity period of the certificate was 1 year, extensions are accordingly made yearly.

Caregivers of all households which were identified as eligible for the USAID Kizazi Kipya program per the program enrollment criteria, and voluntarily accepted to be enrolled in the program, signed an informed consent form after which the enrollment followed. The consent process involved informing caregivers of the project as well as the benefits of enrolling in the project. They were also informed that the project carries no risks, physical harm, pain, or danger. Since the project required the caregivers to voluntarily reveal their HIV status and other personal information, they were assured of zero stigma from project staff and confidentiality and security of information and data. They were also informed that they have the right to terminate enrollment at any time or decide not to participate in certain activities of the program and this will not affect their access to health services or amount into any adverse consequence such as damage to their social status or incur any financial costs. Data submitted electronically has been further protected by ensuring that all tablets are password-protected and the upload/download processes to and from the server are encrypted. Individual data is limited to a few highly trained and experienced monitoring and evaluation staff, and it is de-identified before being shared for analysis.

## Results

### Profile of respondents

The analysis was based on 59,683 caregivers of OVC aged 18 years or more. Their mean and median age was 50.4 years (standard deviation (SD) = 14.8) and 49.0 years (interquartile range (IQR) = 23.0) respectively. These were distributed as: 18.2% (10,861/59,683) HIV positive, 44.6% (26,636/59,683) HIV negative, and 37.2% (22,186/59,683) undisclosed HIV status. Details of the HIV status by region are presented in Additional file 1. The majority of the caregivers were women (71.2%). Forty-four percent of the caregivers were married or living together, 37.3% were widowed, 12.6% were divorced or separated, and 6.1% had never been married. In terms of education, 24.5% had never attended school, 72.5% had primary education, and 3.0% had at least secondary education. Almost two-thirds (64.5%) of the caregivers resided in rural areas and 35.5% resided in urban areas. Close to one-fifth (18.6%) of the caregivers were from food insecure households (Table 1).

**Table 1:** Profile of respondents (n = 59,683)

Variable	Number of respondents (n)	Percent of respondents (%)
All	59,683	100.0
Undisclosed HIV status		
No	37,497	62.8
Yes	22,186	37.2
Caregiver sex		
Female	42,516	71.2
Male	17,167	28.8
Caregiver age (years)		
18–30	4329	7.3
31–60	38,449	64.4
61+	16,905	28.3
Mean = 50.4, SD = 14.8	–	–
Caregivers’ marital status		
Married or living together	26,255	44.0
Divorced or separated	7567	12.7
Never been married	3611	6.1
Widow/widower	22,250	37.3
Caregiver’s education		
Never attended	14,600	24.5
Primary	43,285	72.5
Secondary+	1798	3.0
Place of residence		
Rural	38,476	64.5
Urban	21,207	35.5
Family member has CHF/TIKA card?		
No	49,445	82.9
Yes	10,238	17.1
Caregiver mentally or physically disabled?		
No	57,195	95.8
Yes	2488	4.2
Is the household food insecure?		
No	48,605	81.4
Yes	11,078	18.6

CHF, Community Health Fund; TIKA, *Tiba kwa Kadi*, SD, standard deviation

### HIV status non-disclosure by background characteristics

As presented in Table 2, overall, 37.2% (n = 22,186) of the caregivers did not disclose their HIV status to the USAID Kizazi Kipya program. This proportion was higher in male (40.2%) than in female caregivers (36.0%), and the difference between them was statistically significant (*p *< 0.001). The proportion of non-disclosure was the highest at 47.0% among caregivers who had never attended school, declined to 34.2% among those who had primary education, and further declined to 29.9% among those who had secondary or higher education (*p *< 0.001).

**Table 2:** Undisclosed HIV status among caregivers by background characteristics in Tanzania, 2017 (n = 59,683)

Variable	% of Caregivers that did not disclose their HIV status % (n)	*p* value*
Overall	37.2 (22,186)	–
Caregiver sex		< 0.001
Female	36.0 (15,292)	
Male	40.2 (6894)	
Caregiver age (years)		< 0.001
18–30	33.5 (1450)	
31–60	32.9 (12,629)	
61+	48.0 (8107)	
Caregivers’ marital status		< 0.001
Married or living together	36.3 (9530)	
Divorced or separated	35.1 (2659)	
Never been married	34.3 (1238)	
Widow/widower	39.4 (8759)	
Caregiver’s education		< 0.001
Never attended	47.0 (6858)	
Primary	34.2 (14,790)	
Secondary+	29.9 (538)	
Wealth quintile		< 0.001
Lowest (Q1)	40.7 (5162)	
Second	36.8 (4813)	
Middle	38.9 (4067)	
Fourth	36.8 (4216)	
Highest (Q5)	32.9 (3928)	
Place of residence		0.148
Rural	37.4 (14,379)	
Urban	36.8 (7807)	
Family member has CHF/TIKA card?		< 0.001
No	37.7 (18,645)	
Yes	34.7 (3541)	
Caregiver mentally or physically disabled?		< 0.001
No	37.0 (21,159)	
Yes	41.4 (1027)	
Is the household food insecure?		< 0.001
No	36.7 (17,821)	
Yes	39.5 (4365)	

CHF, Community Health Fund; TIKA, *Tiba kwa Kadi*

*p-values are based on Pearson’s Chi Square test

Non-disclosure also varied significantly by marital status (p < 0.001), whereby 39.4% of the widowed caregivers did not disclose their HIV status. Status non-disclosure was 36.3% among caregivers who were married or living together with their spouses, 35.1% among caregivers who were divorced or separated, and 34.3% among never married caregivers. HIV status non-disclosure was 37.7% among caregivers without health insurance and lower at 34.7% among caregivers with health insurance (*p *< 0.001). The presence of physical or mental disability was associated with a higher non-disclosure rate (41.4%) than among those with no reported disability (37.0%) (*p *< 0.001). Non-disclosure was higher among caregivers from food insecure households (39.5%) than those from food secure households (36.7%) (*p *< 0.001).

### Multivariate analysis

Table 3 presents results from the multivariate analysis, whereby adjusted odds ratios (OR) and their corresponding 95% confidence intervals (CI) of the factors associated with undisclosed HIV status are shown. The likelihood of HIV status non-disclosure was higher by 22% among male than female caregivers (OR = 1.22, 95% CI 1.16–1.28). Similarly, caregivers aged 61 years or more were 72% more likely to not disclose their HIV status than those who were aged 18–30 years (OR = 1.72, 95% CI 1.59–1.88). The likelihood of HIV status non-disclosure was similar between caregivers in the age groups 31–60 years and 18–30 years (OR = 0.96, 95% CI 0.89–1.04). HIV status non-disclosure was also significantly associated with marital status, whereby caregivers who were not married (OR = 1.12, 95% CI 1.03–1.23), and those who were widowed (OR = 1.12, 95% CI 1.07–1.18) were more likely to not disclose their HIV status than those who were married or living together. Caregivers who were divorced or separated were similar with those who were married or living together with their spouses in terms of HIV status non-disclosure (OR = 1.01, 95% CI 0.95–1.08). Rural residence was associated with a 58% more likelihood of HIV status non-disclosure than urban residence (OR = 1.58, 95% CI 1.34–1.86). Lack of health insurance was associated with higher likelihood of HIV status non-disclosure (OR = 1.36, 95% CI 1.28–1.45). Caregivers who were physically or mentally disabled were 14% more likely to not disclose their HIV status than those who were not (OR = 1.14, 95% CI 1.04–1.25).

**Table 3:** Multivariate random-effects logistic regression model of factors associated with undisclosed HIV status among male and female caregivers of orphans and vulnerable children in Tanzania, 2017 (n = 59,683)

Covariate	All (n = 59,683)			Female (n = 42,516)			Male (n = 17,167)		
	Adjusted odds ratio (OR)	95% confidence		Adjusted odds ratio (OR)	95% confidence		Adjusted odds ratio (OR)	95% confidence	
		Lower limit	Upper limit		Lower limit	Upper limit		Lower limit	Upper limit
Caregiver sex									
Female	1.00	–	–	–	–	–	–	–	–
Male	1.22***	1.16	1.28	–	–	–	–	–	–
Caregiver age (years)									
18–30^a^	1.00	–	–	1.00	–	–	1.00	–	–
31–60	0.96	0.89	1.04	1.00	0.91	1.09	0.85	0.72	1.01
61+	1.72***	1.59	1.88	1.87***	1.69	2.07	1.46***	1.22	1.74
Caregivers marital status									
Married or living together^a^	1.00	–	–	1.00	–	–	1.00	–	–
Divorced or separated	1.01	0.95	1.08	1.03	0.96	1.11	0.96	0.84	1.09
Never been married	1.12**	1.03	1.23	1.08	0.98	1.19	1.45***	1.17	1.81
Widow/widower	1.12***	1.07	1.18	1.12***	1.06	1.19	1.10	0.99	1.21
Caregiver’s education									
Never attended school^a^	1.00	–	–	1.00	–	–	1.00	–	–
Primary	0.67***	0.64	0.70	0.67***	0.63	0.71	0.70***	0.63	0.77
Secondary+	0.56***	0.50	0.64	0.54***	0.47	0.63	0.60***	0.49	0.74
Wealth quintile									
Lowest (Q1)^a^	1.00	–	–	1.00	–	–	1.00	–	–
Second	0.92**	0.87	0.99	0.94	0.87	1.01	0.90	0.79	1.03
Middle	0.98	0.92	1.05	1.02	0.94	1.10	0.87**	0.77	0.98
Fourth	0.91**	0.85	0.98	0.98	0.90	1.06	0.74***	0.66	0.84
Highest (Q5)	0.83***	0.78	0.89	0.85***	0.78	0.93	0.73***	0.64	0.83
Place of residence									
Rural^a^	1.00	–	–	1.00	–	–	1.00	–	–
Urban	1.58***	1.34	1.86	1.58***	1.33	1.89	1.22	0.98	1.52
Family member has CHF/TIKA card?									
Yes^a^	1.00	–	–	1.00	–	–	1.00	–	–
No	1.36***	1.28	1.45	1.31***	1.21	1.41	1.41***	1.26	1.57
Caregiver mentally or physically disabled?									
No^a^	1.00	–	–	1.00	–	–	1.00	–	–
Yes	1.14**	1.25	1.04	1.10	0.97	1.25	1.18**	1.02	1.37
Is the household food insecure?									
No^a^	1.00	–	–	1.00	–	–	1.00	–	–
Yes	1.04	0.99	1.1	1.07**	1.00	1.14	0.98	0.88	1.09
Constant	0.32***	0.27	0.38	0.31***	0.26	0.37	0.55***	0.43	0.71
rho (intraclass correlation coefficient)	0.42	0.39	0.45	0.41	0.38	0.44	0.37	0.34	0.41
^a^Reference category	***p < 0001 **p < 005; Number of wards = 995 Number of caregivers per ward: min = 1 avg = 60.0 max = 971; Integration points = 20			***p < 0001 **p < 005; Number of wards = 995 Number of caregivers per ward: min = 1 avg = 43.2 max = 727; Integration points = 20			***p < 0001, **p < 005; Number of wards = 929 Number of caregivers per ward: min = 1 avg = 18.5, max = 294; Integration points = 20		

Education and wealth quintile were protective factors as far as HIV status non-disclosure was concerned. In a dose-response fashion, as education improved, the odds of HIV status non-disclosure declined. Caregivers who had primary education were 33% less likely to not disclose their HIV status than those who had never attended school (OR = 0.67, 95% CI 0.64–0.70). And caregivers who had education of secondary or higher level were 44% less likely to not disclose their HIV status than those who had never attended school (OR = 0.56, 95% CI 0.50–0.64). With respect to economic status, caregivers in the second wealth quintile were 8% less likely to not disclose their HIV status than those in the lowest quintile (OR = 0.92, 95% CI 0.87–0.99). Those in the middle wealth quintile were 2% less likely to not disclose their HIV status, but this was not statistically significant. Caregivers in the fourth wealth quintile were 9% less likely to not disclose their HIV status than those in the lowest quintile (OR = 0.91, 95% CI 0.85–0.98). Finally, caregivers in the highest wealth quintile were 17% less likely to not disclose their HIV status than those in the lowest quintile (OR = 0.83, 95% CI 0.78–0.89). In summary, better education, and higher wealth were associated with a higher likelihood of HIV status disclosure.

The analysis also looked at trends of non-disclosure of caregivers who lived in the same geographic area to see if people in specific areas have non-disclosure clusters, which may imply unmeasured factors that influence non-disclosure at that geographic level. The analysis considered a ward as a cluster. In Tanzania, a ward is an administrative structure with specific government boundaries for a geographic area composed of villages (for rural areas) or streets (for urban areas). Results showed that forty-two percent (42%) of the variability in HIV status non-disclosure among caregivers was due to residence in the same ward (ICC = 0.42, 95% CI 0.39–0.45). In other words, caregiver living in proximity of one another were more likely to behave in a similar way as far as HIV status disclosure was concerned.

## Discussion

This study assessed the socio-demographic factors associated with undisclosed HIV status among caregivers of orphans and vulnerable children (OVC) in Tanzania. Findings showed that overall, 37.2% of the caregivers did not disclose their HIV status to the USAID Kizazi Kipya project during enrollment. Although the project encouraged them to disclose their status, their non-disclosure affected identification and provision of HIV services appropriate for their HIV status. Through services provided by the project especially HIV status disclosure support, the observed disclosure gap at enrollment is expected to ultimately bridge. Non-disclosure of HIV status has been linked with risk behaviors for HIV transmission [19]; in fact evidence from one mathematical modeling analysis which was based on an empirical study of sexual behaviour of HIV positive heterosexual, gay, and bisexual men in Los Angeles, USA [63] has shown up to 41% reduction in HIV transmission attributable to serostatus disclosure [20]. HIV status disclosure may lead to improved access to HIV prevention and treatment services as well as increased opportunity for risk reduction and increased opportunities to plan for the future [19].

This study identified several socio-demographic factors with significant association with undisclosed HIV status. Education was the strongest of the factors, with a dose-response protective effect: the likelihood of HIV status non-disclosure declined as caregivers’ education improved. This may have been due to the role that education plays in enhancing self-esteem, self-confidence, ability to make decisions, and freedom of expression [64], all of which may enable HIV status disclosure. This observation is consistent with one study among HIV positive patients in Kolkatta, India that found higher rates of HIV status disclosure to partners among patients who were literate than illiterate ones [37], as well as in Ethiopia where HIV positive people receiving care and treatment services from an ART clinic were more likely to disclose their HIV status to their sexual partners, family or other members of the population if they had higher level of education [65]. This trend was also observed among HIV positive individuals with tertially education in Nigeria [66]. It is possible that as one’s education improves, communication skills to disclose one’s status improve as well. In Uganda, one study found that communication skills (which has been observed in some studies to be positively associated with education [67, 68]) improves HIV status disclosure [45]. Overall, the fact that education improved caregivers’ HIV status disclosure to a program, suggests the need to continue promoting universal formal education attainment for medium– and longer– term gain in all aspects of the HIV epidemic control, particularly HIV status disclosure.

Similarly, this study found that higher wealth quintiles were significantly associated with lower odds of HIV status non-disclosure. These findings are consistent with previous studies which have found lower disclosure rates among women with economic vulnerabilities and those in low wage-employments [35, 36], though another study did not find similar trends among men [35]. Despite the variation in the findings, economic and social disadvantages may make HIV status disclosure more difficult [24], suggesting that improving the economic situation for people living in high burden HIV environments may also increase status disclosure. As the mechanisms through which economic status is related to HIV status disclosure is still unknown, further research to uncover these pathways is needed.

With respect to sex, male caregivers were 22% more likely to not disclose their HIV status to the program during enrollment than their female counterparts. Men have been reported to be poor health seekers, including HIV testing, compared to women [69, 70], which similarly may explain their lower disclosure rates. A similar observation was made by one systematic review [24]. While men should be targeted with disclosure support interventions, further research is needed to identify the underlying gender-related mechanisms which should be addressed to facilitate disclosure.

Caregiver age and marital status were significantly associated with HIV status non-disclosure to the program during enrollment, and consistent with existing research [40], including in Kenya [38] and Nigeria [39]. This study’s findings also show that both unmarried and widowed status were significantly associated with higher HIV status non-disclosure than married. This was consistent with one study in the Ukraine that found higher non-disclosure among the unmarried [71] and many other studies from sub-Saharan Africa which also found higher disclosure among married or cohabiting respondents than in non-marital unions [5, 38–40]. It may be that adults in marital unions are more likely to disclose to each other for a variety of reasons (e.g. improved communication, trust in each other, etc.) than adults who are not married, and that disclosure to family leads to comfortability disclosing to others, such as community HIV programs.

Furthermore, and consistent with other studies [19, 24, 40] this study observed that HIV status non-disclosure was significantly (58%) more likely in rural areas than in urban areas. This may be due to better access to education, information, health campaigns and health services in urban areas, which can shape residents’ behaviors and attitudes as previously described, leading to increased likelihood of HIV status disclosure. Therefore, rural areas need to be targeted with context-specific efforts including stigma reduction strategies to enhance their HIV status disclosure to HIV treatment and prevention programs for timely access to services.

Physical or mental disability was associated with higher likelihood of non-disclosure. There may be less access to services or information on social and behaviour change communication and the importance of HIV status disclosure due to disability. Additionally, there may be interaction among disabilities, HIV status, and stigma. It has been substantially established that people with disabilities experience discriminatory behaviors from nondisabled persons [72–74], and in fact stigma has been shown to be one of the most important barriers to HIV status disclosure [15–17]. As such, those who are disabled may already be burdened by stigma in their communities, and in order to limit increased stigma from HIV status- whether positive or negative- they prefer to keep their status confidential. Programs and interventions should provide particular support to those with disabilities to improve HIV status disclosure, as well as reducing stigma toward the disabled and PLHIV in the community.

Finally, there was a significant association between lack of health insurance and HIV status non-disclosure. It is possible that caregivers who do not have health insurance are likely to be without good health seeking behaviour, thus less likely to disclose their HIV status to the program. This implies that improving health insurance coverage is likely to increase HIV status disclosure to community-based HIV programs, thus offering a basis for timely service delivery. Unfortunately, there was no related evidence of this association from the literature, thus a need for further research to investigate the underlying mechanisms of the observed association.

### Limitations

This study did not analyze results of caregiver disclosure in relation to HIV status of CCW volunteers because the project does not collect that information. However, we have observed in program implementation that HIV-positive CCWs are living examples of HIV disclosure that can positively influence others in their community to disclose.

## Conclusions

Close to 40% of caregivers of OVC in Tanzania did not disclose their HIV status to the USAID Kizazi Kipya program during enrollment. HIV status non-disclosure was significantly associated with sex, age, marital status, education, wealth quintile, place of residence, health insurance, and physical and/or mental disability. While better education, economic empowerment to address poverty, and expanding health insurance coverage are likely to improve HIV status disclosure, targeted efforts to improve disclosure may be required for men, unmarried, widows, rural areas, the disabled, and elderly populations.

The USAID Kizazi Kipya program provides context-specific direct and indirect services to all members of the enrolled households. For caregivers who disclose their HIV status to the program as HIV positive and not on treatment, escorted referral to initiate the treatment is provided. If the caregiver is already on ART, enquiries about adherence are made, and adherence counselling is made if necessary. Therefore, in view of these results, there is a clear missed opportunity for timely provision of readily available care and treatment services for caregivers that might have been HIV positive as well as linking HIV-negative caregivers and/or at-risk caregivers with prevention services. Further research is needed for better understanding of the underlying mechanisms for these findings.

## Supplementary information

**Additional file 1.** OVC caregivers’ HIV status at enrollment in the USAID Kizazi Kipya project during January–March 2017 in 18 regions of Tanzania (n = 59,683).

## Data Availability

The datasets analyzed during the current study are not publicly available due to confidentiality restrictions, but are available from the corresponding author on reasonable request.

## Abbreviations

AIDS: Acquired immunodeficiency syndrome
ART: Antiretroviral therapy
CHF: Community health fund
CI: Confidence interval
FCAA: Family and child asset assessment
HIV: Human immunodeficiency virus
ICC: Intra-class correlation coefficient
LHIV: Living with HIV
MRCC: Medical Research Coordinating Committee
NIMR: National Institute for Medical Research
OR: Adjusted odds ratio
OVC: Orphans and vulnerable children
PCA: Principal component analysis
PLHIV: People living with HIV
SD: Standard deviation
TIKA: *Tiba kwa Kadi*
UNAIDS: Joint United Nations Programme on HIV and AIDS
USAID: United States Agency for International Development
